# *In-vitro* NMR Studies of Prostate Tumor Cell Metabolism by Means of Hyperpolarized [1-^13^C]Pyruvate Obtained Using the PHIP-SAH Method

**DOI:** 10.3389/fonc.2020.00497

**Published:** 2020-04-17

**Authors:** Eleonora Cavallari, Carla Carrera, Ginevra Di Matteo, Oksana Bondar, Silvio Aime, Francesca Reineri

**Affiliations:** ^1^Department of Molecular Biotechnology and Health Sciences, Center of Molecular Imaging, University of Turin, Turin, Italy; ^2^Institute of Biostructures and Bioimaging, National Research Council, Turin, Italy

**Keywords:** nuclear magnetic resonance, pyruvate, hyperpolarization, para-hydrogen, metabolism

## Abstract

Nuclear Magnetic Resonance allows the non-invasive detection and quantitation of metabolites to be carried out in cells and tissues. This means that that metabolic changes can be revealed without the need for sample processing and the destruction of the biological matrix. The main limitation to the application of this method to biological studies is its intrinsic low sensitivity. The introduction of hyperpolarization techniques and, in particular, of dissolution-Dynamic Nuclear Polarization (d-DNP) and ParaHydrogen Induced Polarization (PHIP) is a significant breakthrough for the field as the MR signals of molecules and, most importantly, metabolites, can be increased by some orders of magnitude. Hyperpolarized pyruvate is the metabolite that has been most widely used for the investigation of metabolic alterations in cancer and other diseases. Although d-DNP is currently the gold-standard hyperpolarization method, its high costs and intrinsically slow hyperpolarization procedure are a hurdle to the application of this tool. However, PHIP is cost effective and fast and hyperpolarized pyruvate can be obtained using the so-called Side Arm Hydrogenation approach (PHIP-SAH). The potential toxicity of a solution of the hyperpolarized metabolite that is obtained in this way is presented herein. HP pyruvate has then been used for metabolic studies on different prostate cancer cells lines (DU145, PC3, and LnCap). The results obtained using the HP metabolite have been compared with those from conventional biochemical assays.

## Introduction

NMR is a powerful and non-invasive tool for the investigation of cellular metabolism as it allows a wide range of metabolites to be detected either in cell cultures or in samples obtained from tissues resected from living systems.

The main drawback of magnetic resonance spectroscopy in biological specimens is the low intensity of the MR signals. Thermal nuclear spin polarization P is typically in the order of 10^−5^-10^−6^ for conventional high-field NMR spectrometers. The signal from water protons is predominant by far, while cell metabolites, whose concentration is about 10,000 times lower, can only be observed *in vivo* with low spatial resolution and after long acquisition times. Moreover, the ^1^H-NMR spectra acquired *in vivo* or in tissues can only provide information about the concentration of metabolites in a steady-state.

The breakthrough in the field came with the introduction of the d-DNP hyperpolarization method (1), which increased the sensitivity of MR signals by some orders of magnitude, thus allowing the visualization of metabolites to be performed in cells and *in vivo*. More interestingly, the acquisition of time-resolved spectra becomes possible, thus providing information about the kinetics of metabolic transformations, in cell cultures and *in vivo* (2, 3).

[1-^13^C]pyruvate is the substrate that has been used most widely for the study of cellular metabolism and its alterations in diseases such as cancer, heart failure and stroke (4–8). Pyruvate plays a central role in cellular metabolism as it can be converted into a number of metabolites, depending on cellular conditions. In most normal tissues, pyruvate dehydrogenase catalyzes the decarboxylation of the pyruvate that enters the TCA cycle. Pyruvate can also be transaminated by alanine aminotransferase or reduced to lactate by lactate dehydrogenase. The presence of disease can alter metabolic fluxes through the different enzymes and an increased glycolytic flux is often observed in tumors. The use of hyperpolarized pyruvate has shown a marked up-regulation in the exchange of the ^13^C hyperpolarized label, mediated by the LDH enzyme, between pyruvate and lactate in tumor cells (9). Hyperpolarized [1-^13^C]pyruvate is currently under intense scrutiny as a potential probe with which to assess the presence and grading of prostate cancer in humans (10).

Unfortunately, d-DNP is an inherently slow, technologically demanding and extremely expensive technique, limiting the application of this powerful tool to a few laboratories worldwide.

ParaHydrogen Induced Polarization (PHIP) allows hyperpolarized substrates to be obtained in a few seconds and with much lower costs than d-DNP (11). This hyperpolarization method is based on the hydrogenation, catalyzed by metal complexes, of an unsaturated precursor of the target molecule (12–14). On this basis the number of PHIP-polarizable substrates appears to be limited by the availability of the proper unsaturated precursor and the concerns about the biological applications of the obtained hyperpolarized products are related to the presence of residual metal catalyst and other impurities that are associated to the hydrogenation reaction. The PHIP- side-arm hydrogenation (PHIP-SAH) approach (15) is a good step ahead as it circumvented both issues and can provide aqueous solutions of hyperpolarized pyruvate, or other metabolites (16), that can be safely used for *in-vivo* and *in-cells* studies (17, 18). Nevertheless, although the PHIP-SAH procedure allows most of the toxic compounds to be removed (catalyst and solvent), traces that may affect the viability and metabolism of cells may still be present in the aqueous phase of the final product.

This work thoroughly investigate the toxicity of aqueous solutions of the HP metabolites by performing *in-vitro* cytotoxicity analyses on prostate cancer cell lines.

HP [1-^13^C]pyruvate was found to be a sensitive tool for investigation into the difference in the metabolic phenotype of two highly aggressive and metastasizing human prostate carcinoma cell lines, PC3 and DU145 and another, less aggressive one, LNCaP.

## Materials and Methods

### [1-^13^C]pyruvate Hyperpolarization

**Figure 1:**
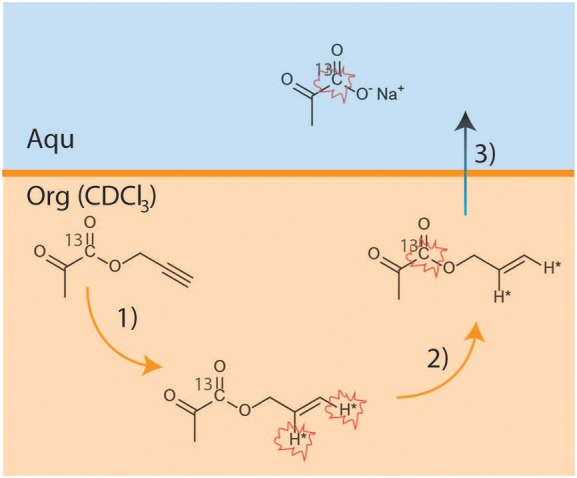
Scheme reporting the key steps of the PHIP-SAH procedure: (1) hydrogenation using parahydrogen of propargyl-[1-^13^C] pyruvate; (2) polarization transfer to the ^13^C carboxylate spin by means of magnetic field cycle (MFC); (3) hydrolysis and phase transfer of the Sodium salt from the organic to the water phase.

^13^C-labeled HP pyruvate was obtained using the PHIP-SAH method (Figure 1). The hydrogenation catalyst ([1,4-bis(diphenylphosphino)butane](1,5-cyclooctadiene) rhodium(I) tetrafluoroborate, Sigma Aldrich, 1.38 μmol) was dissolved in deuterated chloroform (CDCl_3_, 100 μl). The propargylic ester of [1-^13^C]pyruvate (3 μl, 26.6 μmol) was added to this solution. A 5 mm NMR tube (equipped with a gas valve) was used as the hydrogenation reactor and was pressurized with 2.1 bar of para-enriched hydrogen (~86% enriched), while the NMR tube was kept in a liquid nitrogen bath.

The sample was kept frozen in liquid nitrogen (time intervals from 0.5 to 7 h) until the start of the hyperpolarization experiment. The hyperpolarization procedure consisted of the following steps:

- Parahydrogenation reaction: the NMR tube containing the frozen reaction mixture was quickly heated, via immersion in a hot water bath (at 353 K for 7 s), and then shaken for 3 s;
- Polarization transfer: the NMR tube was opened to release the parahydrogen pressure and immediately placed in the magnetic field shield, to which magnetic field cycling (MFC) was applied (18).
- Hydrolysis: a base solution (260 μl of NaOH 0.1 M and Sodium Ascorbate 50 mM), pressurized using argon (2bar) and heated at ~353 K, was injected into the organic solution to hydrolyze the hyperpolarized allyl-ester. An acidic buffer solution (100 μl HEPES 144 mM, pH 5.4) was then added to the aqueous phase to reach physiological pH (7.2 ± 0.2 was measured).
- Phase separation: the aqueous solution of the HP product (250 ul) was taken up into a syringe and diluted with 600 μl of distilled H_2_O before being transported to the NMR spectrometer, where 230 ± 11 ul of this solution was quickly added to the NMR tube containing the cell suspension.

The concentration and hyperpolarization of [1-^13^C]pyruvate was measured at the end of each experiment by acquiring a ^13^C-NMR spectrum of the thermally polarized product with the addition of ^13^C-urea as an internal reference. The pyruvate concentration in the cell suspensions was found to be 5.0 ± 0.4 mM.

### ^13^C-NMR Experiment Set-Up

The ^13^C-NMR spectra were carried out using 5 mm NMR tubes, instead of the previously reported 10 mm sample tubes (18). A standard 5 mm-OD Wilmad NMR tube was equipped with a cap modified with a central hole (inner diameter 2 mm) and a glass tube (2 mm OD, 1.75 mm ID, 8 mm L) was inserted into the cap. A PTFE tube (1 mm OD, 0.75 mm ID, 750 mm L) was placed inside the glass capillary tube and kept in the axial position, a few mm above the cells suspension. The quick injection of the aqueous solution of the hyperpolarized substrate through the PTFE injection line allowed the cell suspension to mix with the HP pyruvate. The PTFE tube was removed immediately after the addition of the HP-pyruvate in order to avoid B_0_ inhomogeneities during the acquisition of the ^13^C-NMR spectra.

### ^13^C-NMR Experiments: Cells Preparations

The metabolic transformation of [1-^13^C]pyruvate, hyperpolarized through the PHIP-SAH procedure, into [1-^13^C]lactate, was assessed on the following specimens: (I) prostate cancer cells suspended in their growth medium; (II) intact cells suspended in lactate-enriched medium; (III) lysed cells. The samples (volume of 300 ul) were placed in the 5 mm NMR tube equipped with the Teflon transfer line ready to receive the addition of the HP substrate. The NMR tube was positioned into the NMR spectrometer (600 MHz Bruker Avance) where it was kept at 310 K.

The contents of the specimens were as follows:

- Pyr/Lac tests in cells suspension: cells were harvested from plates (2 ml of warm 0.25% Trypsin 0.53 mM EDTA solution), counted using a Burker chamber and then centrifuged in the growth medium for 5 min at 125 × g-force. Once the supernatant was removed, 9.2 ± 0.2 M cells for each experiment were found to be suspended in 300 μl of culture medium and transferred into a 5 mm NMR test tube.
- Pyr/Lac test in cells suspension in the presence of added Lac: the cell-containing sample was prepared as in I, L-lactate was then added to the culture medium and left to equilibrate for 10 min in the NMR spectrometer before the injection of the HP-pyruvate. At the end of the experiment, after the addition of the aqueous solution of the HP metabolite, the final lactate concentration was 5.94 ± 0.14 mM.
- Pyr/Lac test in the presence of Lysed cells: for each experiment, the lysed cell solution was obtained by twice freeze-thawing cell suspension in liquid nitrogen. The sample-containing cells were prepared as in II. In this set of experiments L-lactate (10 mM) was also added to the growth medium and left to equilibrate for 10 min in the NMR spectrometer before the HP experiment.

The acquisitions of the ^13^C-NMR spectra were carried out using a small flip angle (18°). A 2 s delay was applied between successive acquisitions. The first acquisition started a few seconds before the injection of the HP substrate.

The vitality of the cells at the end of the hyperpolarized experiment was checked using the trypan blue exclusion test. The viability in all of the experiments was 95%.

At the end of the experiment the number of cells was checked via the quantification of Bradford proteins, using the specific calibration line for each cell line.

The ^13^C NMR spectra were acquired on a Bruker Avance 14.1 T NMR spectrometer using a 5 mm BBO probe equipped with ^1^H and ^13^C coils.

### Cells Cultures

The PC3, DU145, and LNCaP (prostate carcinoma) cell lines were purchased from American Type Culture (ATCC®). Cells were kept for 72 h in 175 cm^2^ flasks at 310 K in a humidified atmosphere with 5% CO_2_ in the recommended culture media as suggested by ATCC, in particular: LNCaP: RPMI1640 (D-glucose 25.0 mM); PC3: F-12K (D-glucose 7.0 mM); DU145: EMEM (D-glucose 5.6 mM). All the cells were used within the first 10 passages from unfreezing.

### *In-vitro* Cytotoxicity

To assess the adverse effects of the aqueous PHIP-SAH hyperpolarization derived solutions, the MTT test (based on the enzymatic reduction of the tetrazolium salt MTT [3-(43-dimethylthiazol-2-yl)-2,5-diphenyl-tetrazoliumbromidein]) in living, metabolically active cells was carried out on two prostate cancer cell lines (DU145 and PC3).

For these tests, cells were harvested via trypsinization, resuspended in fresh medium, and plated in the wells of 96-well-microtiter plates at a volume of 0.1 ml. Routinely 7,000–16,000 cells, depending on the cell-lines growth curves, were plated in each well. After 24 h, to ensure the cells adhesion, the culture medium was removed, and replaced with a fresh medium of the same composition as the one used in the hyperpolarization procedure, i.e., 15% of the aqueous solution in the final volume (0.1 ml).

At the end of the incubation period (1, 6, and 24 h), the medium was replaced with 0.1 ml of a 5 mg/ml solution of MTT (purchased from Sigma, St. Louis, MO) in phosphate-buffered saline (PBS 1X). After 4 h of incubation with MTT at 310 K, the supernatant was carefully sucked off and a solubilization solution, 0.15 ml of dimethyl sulfoxide, was added to dissolve the insoluble purple formazan product into a colored solution and the absorbance at 570 nm was read by a spectrophotometer.

Cells were plated in triplicate to minimize the variability of the results. In each plate, 3 control wells for each cell line were included.

The PC3 and DU145 cells were treated with the following solutions:

1. Aqueous solution of the product of the PHIP-SAH procedure, prepared as described in the [1-^13^C]pyruvate hyperpolarization paragraph (i.e., the aqueous phase resulting after step c).
2. Aqueous solution obtained from the injection of the pressurized and heated base through a chloroform solution of the hydrogenation catalyst, without the addition of propargyl-pyruvate. The solution is analogous to that in 1 but is expected to contain only residues of chloroform and catalyst impurities.
3. Aqueous solution obtained from the injection of the pressurized and heated base through chloroform, without the hydrogenation catalyst. The aqueous solution is expected to contain only traces of chloroform.
4. Aqueous solution of allyl-alcohol, at the same concentration as that obtained from the hyperpolarization procedure.
5. Aqueous solution of the product of the PHIP-SAH procedure, prepared with the addition of ethanol (5 ul) to the organic phase. In this case the composition of the aqueous solution is analogous to that in 1, but with an excess of ethanol. As this composition was the one used in a previous *in-vivo* study (17), its toxicity was tested in this work for completeness.

### Lactate Dehydrogenase Assay

A commercial kit (Sigma-Aldrich MAK066) was used to measure the LDH activity in the cell lines. The kit was used according to the manufacturer's instructions. Briefly, 1 × 10^6^ cells per sample were rapidly homogenized by sonication (30% power, 21 W, for 30 s) over ice in 500 μl of cold LDH Assay buffer, and was then centrifuged at 10,000 g for 15 min at 277 K to remove the insoluble material.

To ensure the readings were within the linear range of the standard curve, 4–10 μl samples were added into duplicate wells of a 96-well-plate, bringing the sample to the final volume of 50 μl with LDH Assay Buffer. After the addition of 50 μl of the Master Reaction Mix the NADH production kinetics were measured via the absorbance of the specific probe at 450 nm.

### Extracellular Lactate Assessment

To determine the concentration of lactate in the supernatant, cells were seeded on 75 mm^2^ plates. After overnight adhesion in standard conditions (310 K and 5% CO_2_), the culture medium was replaced with a new one. After 72 h, the medium was collected, and the cells were harvested by trypsinization and then counted. The applied procedure allowed the amount of lactate measured in the culture medium to be normalized to the number of cells.

In order to immediately quench any possible residual metabolism, one volume of culture medium was mixed with two volumes of cold methanol in a vial, snap-frozen and left 2 h in liquid nitrogen. Proteins were then allowed to precipitate at 250 K for 30 min and the sample was centrifuged at 16,000 g at 277 K for 20 min. The supernatant was then collected and immediately lyophilized to remove the methanol for subsequent measurements, and was then reconstituted with 0.6 ml of phosphate buffer (0.15 M K_2_HPO_4_, pH 7.0) in deuterated water (D_2_O) for ^1^H NMR quantification.

The amount of extracellular lactate was determined using NMR ^1^H spectrometry (see Supplementary Material). Experiments were carried out in triplicate and the lactate concentration in the samples (μmol/cell) was calculated based on the internal standard reference. A known amount (0.35 mM) of 3-(trimethylsilyl)-propionic-d_4_ acid sodium salt (TSP-d_4_) was added to act as a chemical shift reference for the calibration of the NMR data (at 0.0 ppm) as well as an internal standard for quantitation.

### Intracellular Lactate Concentration

Methanol-chloroform-water extraction (M/C) was used to extract metabolites from cells (19).

Briefly, cell pellets were promptly quenched in liquid nitrogen followed by the addition of 0.5 ml of cold methanol-chloroform solution at a ratio of 2:1. After thawing over ice, samples were vortexed for 60 s and sonicated. After 15 min of contact with the M/C solution, 0.25 ml of chloroform and 0.25 ml of distilled water were added to the mixture to yield an emulsion, which was vortexed and centrifuged at 13,000 g for 20 min at 277 K. The upper layer which contained the water-soluble metabolites was collected, lyophilized and reconstituted with 0.6 ml of phosphate buffer (0.15 M K_2_HPO_4_, pH 7.0) in deuterated water (D_2_O) for ^1^H NMR quantification (see Supplementary Material).

An internal standard (TSP-d_4_) concentration of 0.03 mM was used as the reference.

## Results

### Biochemical Assays

#### Cytotoxicity

**Figure 2:**
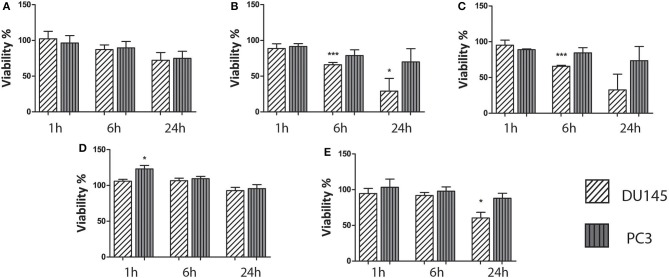
Results on the biocompatibility of the aqueous solutions obtained from the PHIP-SAH procedure. All cell lines were treated with: **(A)** an aqueous solution of pyruvate obtained from the PHIP-SAH procedure; **(B)** an aqueous solution containing traces of chloroform and catalyst; **(C)** an aqueous solution containing chloroform traces; **(D)** an aqueous solution of allyl alcohol and **(E)** the aqueous solution of the product of the PHIP-SAH procedure, to which ethanol was added. The cells viability was assessed by MTT assay. Data are the mean +SD from three independent experiments (*n* = 3). *P ≤ 0.05, ***P ≤ 0.001, ns: not significant vs. untreated control (100% viable); one-way ANOVA.

The cytotoxicity of the aqueous HP-pyruvate-containing solutions obtained by PHIP-SAH (solution I) and of single components was assessed. Cell viability was not affected by solution I after 1 and 6 h of treatment while a toxicity effect was observed after 24 h (Figure 2A). The solutions that contained traces of chloroform plus catalyst (solution II) and chloroform alone (solution III), showed a significant effect on the cell viability after 6 h of treatment (Figures 2B,C). As their toxicity appeared to be the same, one can draw the conclusion that it is mainly associated with the presence of the chloroform traces in the water phase. The hydrolysis side product allyl alcohol (solution IV) did not show any significant effect on the cell viability (Figure 2D). The presence of ethanol, the hydrogenation co-solvent, in the water solution, had a toxicity effect after 24 h treatment (Figure 2E).

#### LDH Activity

**Figure 3:**
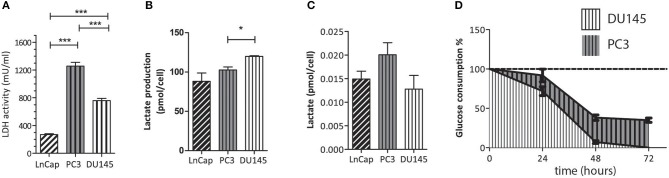
**(A)** LDH activity in the three cell lines measured using a conventional biochemical assay on cells lysates; **(B)** amount of extracellular lactate obtained from ^1^H-NMR spectra of the growth medium; **(C)** amount of intracellular lactate measured by means of ^1^H-NMR of cells lysates; **(D)** rate of glucose consumption in PC3 and DU145 cells as obtained from the ^1^H-NMR spectra of the growth medium at three different timepoints. *P ≤ 0.01, ***P < 0.001; unpaired t-test.

The conventional biochemical assay used for the measurement of LDH activity showed that this enzyme was significantly more active in PC3 than in DU145 cells (Figure 3A), while in the LNCaP cells the activity of LDH was significantly lower than in both the other. This latter finding is in agreement with the fact that LNCaP cells are less glycolytic than the other two, more aggressive prostate tumor models. It has already been shown that the metabolic phenotype of the LNCaP cells is dramatically different from that of PC3 and DU145 cells (20). The difference of LDH activity between PC3 and DU145 cells is also significant, being PC3 cells much more glycolytic than DU145 cells. This difference has been further investigated by means of the metabolomic measurements carried out in growth media and cells extracts.

#### ^1^H-NMR Metabolomic Measurements

From the ^1^H-NMR spectra of the culture medium it was found that the production and accumulation of lactate in the extracellular space is slower in the less aggressive cells (LNCaP). If only the other two, more aggressive cell lines are considered, the lactate production is faster in DU145 than in PC3 cells (Figure 3B). Conversely, the intracellular lactate pool was larger in PC3 than in DU145 cells (Figure 3C).

Measurements of the glucose content in the extracellular medium, at different timepoints, showed that the rate of glucose consumption was faster in DU145 than in PC3 cells (Figure 3D). These results would appear to imply that glycolytic efficiency is higher in PC3 than in DU145 cells.

### ^13^C-Hyperpolarization Experiments

**Figure 4:**
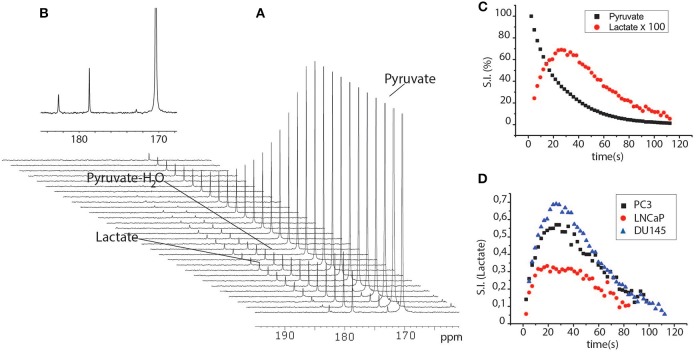
**(A)** series of ^13^C-NMR spectra acquired after the perfusion of a cells suspension (PC3 cells, 10 M) with the aqueous solution HP-[1-^13^C]pyruvate. Spectra were acquired using small flip angle pulse (18°) and 2 s delays between one scan and another. **(B)** expanded ^13^C-NMR spectrum at maximum intensity of the lactate signal; **(C)** Time dependent pyruvate and lactate curves obtained from the integrals of the signals of the two metabolites in the ^13^C-NMR spectra reported in **(A)**. **(D)** time dependent lactate curves obtained after the addition of HP-pyruvate to a suspension of DU145, PC3, and LNCaP cells.

When HP-[1-^13^C]pyruvate was added to the cells suspended in their growth medium and then kept in the NMR spectrometer at physiological temperature, lactate signal build-up was observed immediately in the ^13^C-NMR spectra series. This was due to the rapid exchange of the ^13^C hyperpolarized label between pyruvate and lactate that occurs in the intracellular compartment (Figure 4). The lactate signal reached a maximum at about 20 s, and then decayed due to the T_1_ relaxation processes. The ^13^C signals that correspond to pyruvate and lactate were integrated and the time-dependent signal intensities of lactate and pyruvate were interpolated using a set of functions in order to obtain information about the kinetics of the metabolic process. The experimental results can be interpolated using a four-pool model that takes into account intra- and extra-cellular pyruvate and lactate, all mutually exchanging (21, 22). However, in the present case, the spectral resolution did not allow us to discriminate between the intra and extracellular metabolite signals, meaning that the two-compartments model was used in order to avoid errors that might be caused by over-parametrization (23).

The observed pyruvate and lactate peak intensities can be fitted to a simple two-sites exchange model

$$\begin{array}{l} Pyr⇌Lac \end{array}$$

according to which, the ^13^C-NMR signals of [1-^13^C]pyruvate and [1-^13^C]lactate are described by the coupled differential equations (24)

(1) $$\begin{array}{l} \frac{dPyr}{dt}=-k_{PL}Pyr\left(t\right)+k_{LP}Lac\left(t\right)-\frac{1}{T_{1P}}Pyr\left(t\right) \end{array}$$

(2) $$\begin{array}{l} \frac{dLac}{dt}=+k_{PL}Pyr\left(t\right)-k_{LP}Lac\left(t\right)-\frac{1}{T_{1L}}Lac\left(t\right) \end{array}$$

where k_PL_ and k_LP_ are the kinetic constants of the pyruvateto-lactate conversion and the T_1_ values are the decay time constants of the ^13^C carbonyl sites. It has been shown that the model can be further simplified by setting the back-conversion rate (k_LP_) to zero (25). It follows that one deals with a two compartment model characterized by an unidirectional flow with the kinetic constant k_PL_ being an apparent overall pyruvate to lactate conversion rate. The solutions of equations 1 and 2, given that Pyr(*t* = 0) = [Pyr]^*^Z, where [Pyr] is the concentration of hyperpolarized pyruvate added to the test tube, Z is the enhancement factor, and Lac(*t* = 0) = 0, are

(3) $$\begin{array}{l} Pyr=Pyr\left(t_{0}\right)\cdot exp\left(-\left(k_{PL}+\frac{1}{T_{1P}}\right)\cdot t\right) \end{array}$$

(4) $$\begin{array}{l} Lac=\frac{T_{1}^{Lac}\;k_{PL}\;Pyr\left(t_{0}\right)\;}{T_{1}^{Lac}\left(k_{PL}+\frac{1}{T_{1}^{Py}}\right)-1}(\exp\left(-\frac{t}{T_{1}^{Lac}}\right)\\ -\exp\left(-\left(k_{PL}+\frac{1}{T_{1}^{Py}}\right)t\right)) \end{array}$$

We must also be consider that the small flip angle pulses (φ) that were used lead to further loss of polarization. In order to take into account this factor, an envelope function exp(−λ*t*) was added to the observed signal intensity, where $\lambda =\frac{ln\left(cos\varphi \right)}{\Delta t}$ and Δt is the time interval between successive pulses (26)

(5) $$\begin{array}{l} Pyr^{\prime }=Pyr.\exp\left(-\lambda t\right) \end{array}$$

(6) $$\begin{array}{l} Lac^{\prime }=Lac.\exp\left(-\lambda t\right) \end{array}$$

The kinetic constants obtained from the fittings were normalized to the number of cells and the pyruvate-to-lactate conversion rate was calculated as follows

$$\begin{array}{l} v_{PL}=\;\frac{k_{PL}{.\left[Pyr\right]}_{t0}.v}{n\;cells} \end{array}$$

where *v* is the volume of the sample.

**Figure 5:**
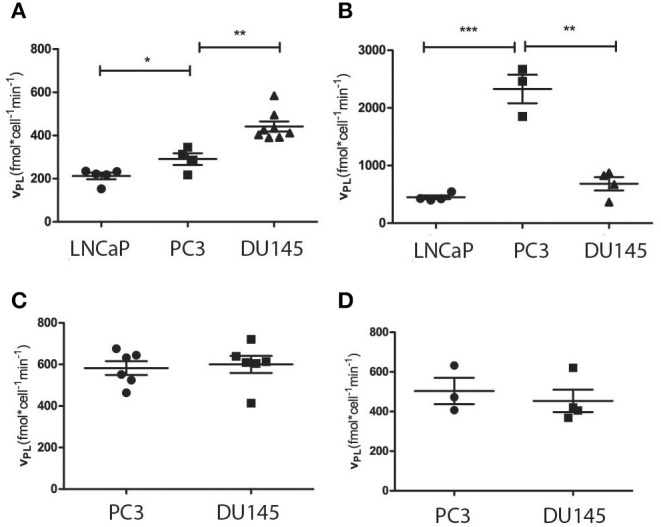
Rate of pyruvate to lactate conversion obtained from the experiments carried out using HP [1-13C]pyruvate on cells, in different conditions: **(A)** intact cells (DU145, PC3 and LNCaP cells) cultured in their proper culture medium, **(B)** intact cells suspended in the medium with added lactate; **(C)** lysed cells and **(D)** cells cultured in the same medium (DMEM). *P ≤ 0.05, **P ≤ 0.01, ***P ≤ 0.001; unpaired t-test.

From the interpolation of the ^13^C-NMR time-dependent signals LNCaP cells resulted to be less glycolytic than the other two (PC3 and DU145), in agreement with the fact that LNCaP cells have a more oxidative metabolic phenotype than the other two (20), while DU145 were significantly more glycolytic than PC3 cells (Figure 5A).

In the second set of experiments, lactate was added to the medium in which cells were suspended, during the 13C-NMR experiment, as reported by Day et al. (27). In this case, the pyruvate-to-lactate conversion rate become significantly faster in PC3 than in DU145 cells, while the apparent glycolytic efficiency of the LNCaP cells was still significantly lower than in the other two cell lines (Figure 5B).

The observed pyruvate-to-lactate exchange rate in intact cells is the result of concomitant processes, namely the rate of metabolite transport through the cellular membrane, and the efficiency of the LDH enzyme. In order to get more information about the differences in the metabolic phenotype of the two, more aggressive cell lines (PC3 and DU145), another series of experiments has been carried out on lysed cells. In these experiments, the contribution of MCT1 mediated transport of pyruvate to the extra to the intracellular compartment was removed, while lactate was added to the medium in order to mimic its intracellular concentration. In these experiments, the exchange kinetics became higher in both cell lines (PC3 and DU145) and were still slightly, even if not significantly, faster in DU145 than in PC3 cells (Figure 5C).

It must also be noticed that the difference between PC3 and DU145 cells was not significant when the cells were maintained in the same growth medium (DMEM) (20) (Figure 5D), while the growth rate of LNCap cells was exceedingly low.

## Discussion

One of the main concerns with the application of parahydrogen hyperpolarized substrates in metabolic studies, both in cells and *in vivo*, is the presence of toxic impurities in the aqueous solution of the HP metabolite, traces of hydrogenation catalyst, organic solvent, and reaction side-products. The cytotoxicity experiments carried out in this work indicate that the aqueous solution of the HP substrate has moderate toxicity when cells are incubated with the product solution for 24 h. Interestingly, the toxicity appeared to be mainly caused by traces of organic solvent (chloroform) while the hydrolysis side-product allyl-alcohol did not affect cell viability after 24 h of incubation. Most importantly, experiments carried out using hyperpolarized substrates usually take place in a timeframe of a few minutes after the perfusion of the HP substrate. Therefore, on the basis of the herein reported results, it can be reasonably assumed that cellular metabolism is not affected by these chemicals during the time course of the experiment.

In this work, the experimental set-up and procedure for in-cell studies has been modified, compared to the previously reported protocol (18). The improved system for the addition of the hyperpolarized product through the cell suspension allowed us to use small size NMR tubes (5 mm NMR tubes) instead of the previously reported 10 mm NMR tubes. The use of a smaller volume is a significant improvement as the amount of cells necessary for each experiment was drastically reduced, from 20 to 8–10 M, and the spectral resolution was also considerably increased.

When cellular metabolism was interrogated using HP-[1-^13^C]pyruvate in intact cells, the pyruvate to lactate conversion rate was slower in LNCaP than in the other two cell lines. This is in agreement with the fact that the metabolic phenotype of LNCaP is significantly different from that of the other two cell lines and is also coherent with the lower LDH activity measured using the conventional biochemical assay. Differently, the pyruvate to lactate conversion rate of PC3 and DU145 cells, measured using hyperpolarized pyruvate, was contradictory with the LDH activity obtained from the biochemical assay applied to both cell lines. In fact, while in the first set of experiments, DU145 appeared more glycolytic than PC3 cells (Figure 5A), the second showed a higher LDH activity in the PC3 cells (Figure 3A).

In order to clarify this apparent contradiction, other series of experiments using HP-[1-^13^C]pyruvate have been carried out on intact cells suspended in their growth medium with added lactate and on lysed PC3 and DU145 cells.

When lactate was added to the extracellular medium of the intact cells, the apparent pyruvate-to-lactate exchange rate was significantly increased in all the cell lines and in particular in the PC3 cells. (Figure 5B).

Conversely, in the experiments carried out on lysed cells, there was not a significant difference between the ^13^C label exchange kinetics of these two cell lines (Figure 5D).

In order to account for these experimental observations, two different hypotheses can be put forward, one relying on the oxidative lactate metabolism and the other based on the MCT4 mediated transport of lactate through the cellular membrane.

The first is based on the well-known fact that different isoforms of LDH exist, that are tetramers of two kinds of subunits (the M type and the H type), and have different affinity either for pyruvate or for lactate. These subunits are encoded by two similar genes (LDH-A and LDH-B, respectively). While LDH-A supports the ability of malignant cells to convert pyruvate into lactate, LDH-B is related to the oxidative use of lactate (28). Therefore, the faster ^13^C label exchange between pyruvate and lactate observed in the PC3 cells, after the addition of lactate to the extracellular medium, might be due to higher LDH-B expression, that leads to the more efficient oxidation of lactate to pyruvate.

The biochemical assays seemed also to support this hypothesis. In fact the biochemical LDH assay measured the rate of lactate-to-pyruvate conversion, through the spectrophotometric observation of NADH formation, and the LDH activity was higher in PC3 than in DU145 cells. From the glucose measurements carried out on the extracellular medium it was also evident that glucose consumption and lactate production was more efficient in DU145 cells, which accumulate lactate in the extracellular compartment. On the other hand, PC3 cells showed smaller glycolytic efficiency and a larger amount of lactate in the intracellular space, which may imply a more efficient use of lactate rather than glucose as an oxidative substrate.

All of these observations seemed to point toward the view that the oxidation of lactate into pyruvate is more efficient in PC3 cells, while the LDH enzyme works preferentially as a pyruvate reductase in the DU145 cells. The oxidative lactate metabolism, associated with MCT-1 facilitated lactate uptake, is at the core of a metabolic adaptation of cancer cells called metabolic symbiosis (28).

The other possible explanation takes into account the effect of MCT-mediated transport on the pyruvate-to-lactate exchange rate, that has previously been investigated using hyperpolarized pyruvate in cells (25, 29, 30) and *in vivo* (31). MCT-1 has a broader distribution and has been associated with the uptake and efflux of pyruvate, L-lactate and others through the plasma membrane, while MCT-4 are mostly associated with the export of lactate in cells with high glycolytic rates (32, 33). The effect of the overexpression of MCT-4 in cancer cells on the pyruvate-to-lactate exchange rate observed using hyperpolarized pyruvate had been investigated as well (23). A recent study on MCT4 carried out by Contreras-Baeza et al. (34) showed that MCT4 is a high-affinity lactate transporter with a somewhat lower affinity for pyruvate. This property confers MCT-4 expressing cells the ability to export lactate against high ambient lactate level, thus maintaining their lactate producing role, while MCT1 cells revert from lactate producers to consumers. A higher expression of MCT4 in DU145 cells than in PC3 may also explain the observation that the pyruvate-to-lactate exchange rate becomes more marked in the former cell line when the concentration of lactate in the extracellular medium is increased. It must also be noticed that the addition of lactate to the medium, in the experiments carried out on lysed cells (Figure 5D), did not lead to any significant difference in the exchange rate.

Although a thorough investigation of the expression of the different isoenzymes (LDHA and LDHB) and MCTs (MC1 ad MCT4) in the two, more aggressive prostate cancer cell lines (PC3 and DU145) would be needed, it can be concluded that the experiments reported herein have shown that the polarization obtained on [1-^13^C]pyruvate, as obtained using the PHIP-SAH methodology is more than sufficient for investigation of the differences in the metabolic phenotype of prostate cancer cells characterized by different aggressiveness (LNCaP, PC3, and DU145). These findings pave the way for a number of possible NMR investigations of cellular metabolism that go well-beyond the pyruvate/lactate transformation investigated in this work.

## Data Availability Statement

The raw data supporting the conclusions of this article will be made available by the authors, without undue reservation, to any qualified researcher.

## Author Contributions

The study was conceived and designed by EC and FR, implemented with the help of CC and OB. CC synthesized the substrates and optimized the preparation of the aqueous solution of the HP product. EC and FR carried out the analysis of the data from hyperpolarized experiments. GD and EC performed the biochemical assays and metabolomic measurements. SA provided conceptual advices on the study. FR, SA, and EC wrote the manuscript. All the authors contributed to the discussion of the results, revised, and approved the final version of the manuscript.

## Conflict of Interest

The authors declare that the research was conducted in the absence of any commercial or financial relationships that could be construed as a potential conflict of interest.
