# Supplementary material for: In-vitro NMR Studies of Prostate Tumor Cell Metabolism by Means of Hyperpolarized [1-13C]Pyruvate Obtained Using the PHIP-SAH Method
Source: Front Oncol. 2020 Apr 17;10:497. doi: 10.3389/fonc.2020.00497 (PMC7180174; doi:10.3389/fonc.2020.00497)
Supplement: Supplementary file 1 [file Data_Sheet_1.PDF]

Supplementary Material

1 Supplementary Figures: extra- and intra-cellular lactate assessment

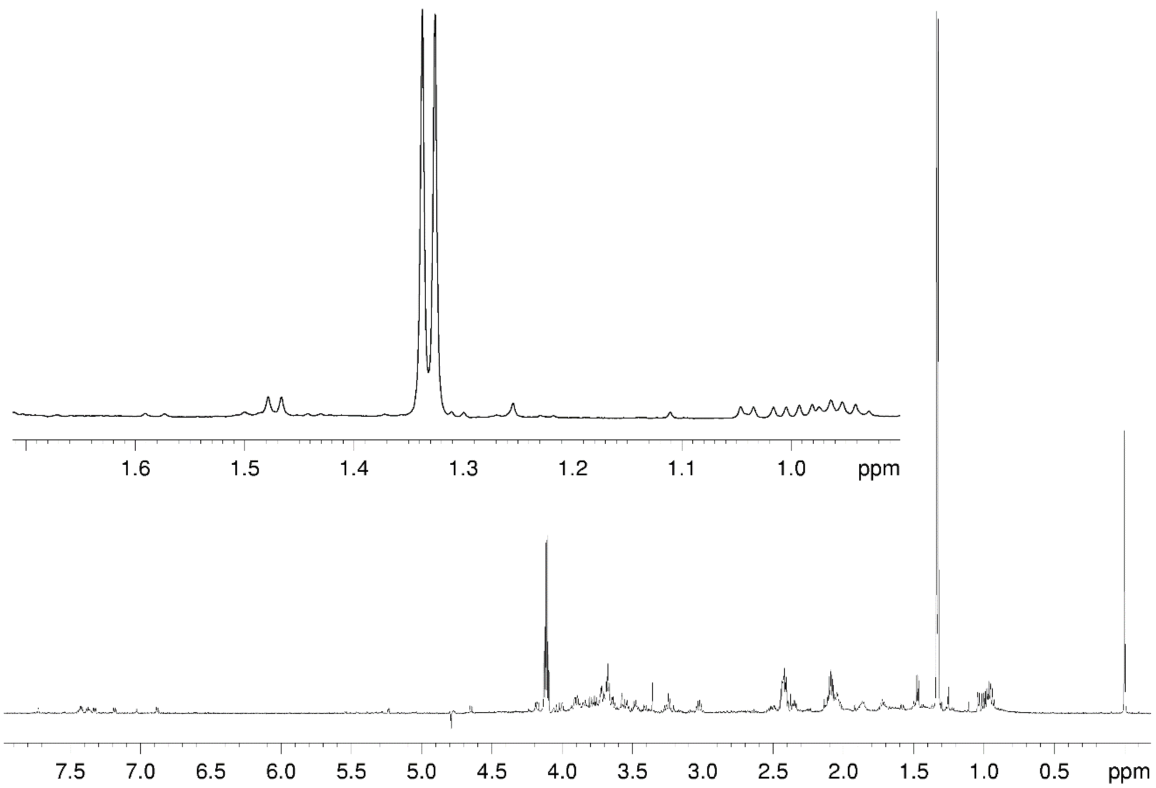

Supplementary Figure 1. 1D ^{1}H NMR spectra of extracellular metabolites from DU145 cells cultured in this medium for 72 hours. The 1.33 ppm signal is the one originating from the methyl lactate protons. At 0.0 ppm the 3-(trimethylsilyl)-propionic-d_4 acid sodium salt (TSP-d_4) protons (0.35 mM).

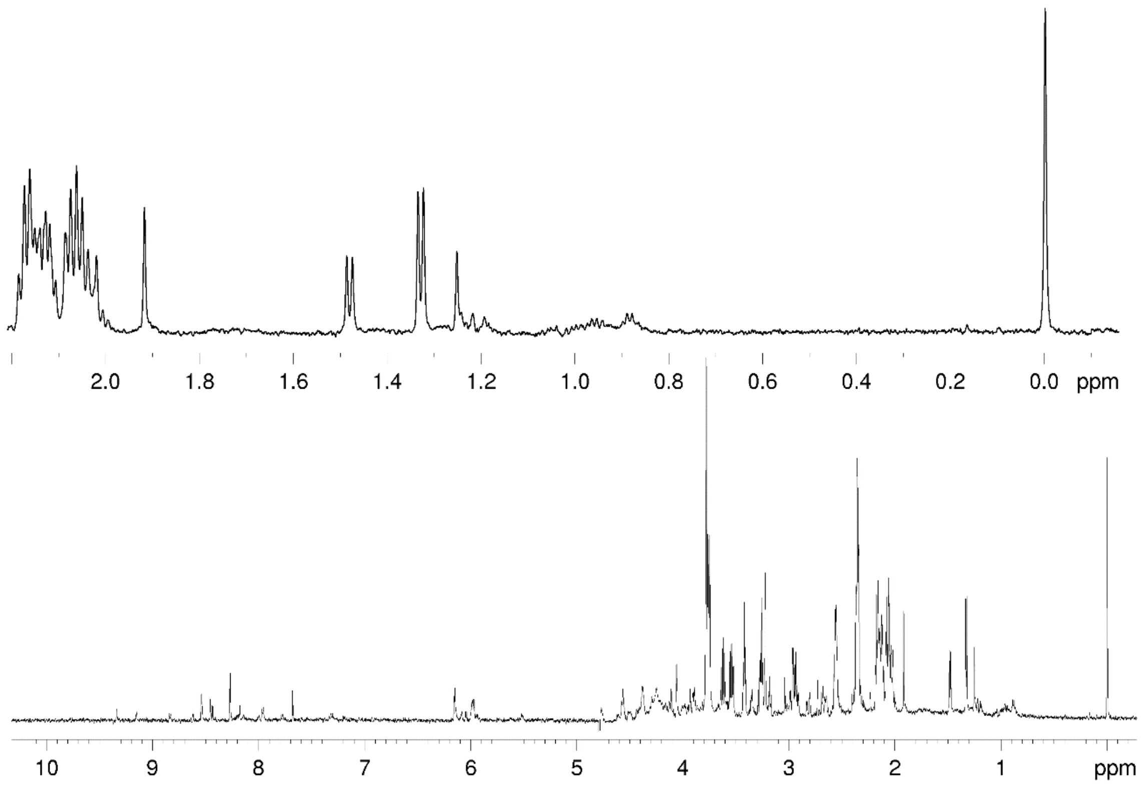

Supplementary Figure 2. 1D ^{1}H NMR spectra of intracellular metabolites from DU145 cells cultured for 72 hours. The 1.33 ppm signal is the one originating from the methyl lactate protons. At 0.0 ppm the 3-(trimethylsilyl)-propionic-d_4 acid sodium salt (TSP-d_4) protons (0.03 mM).

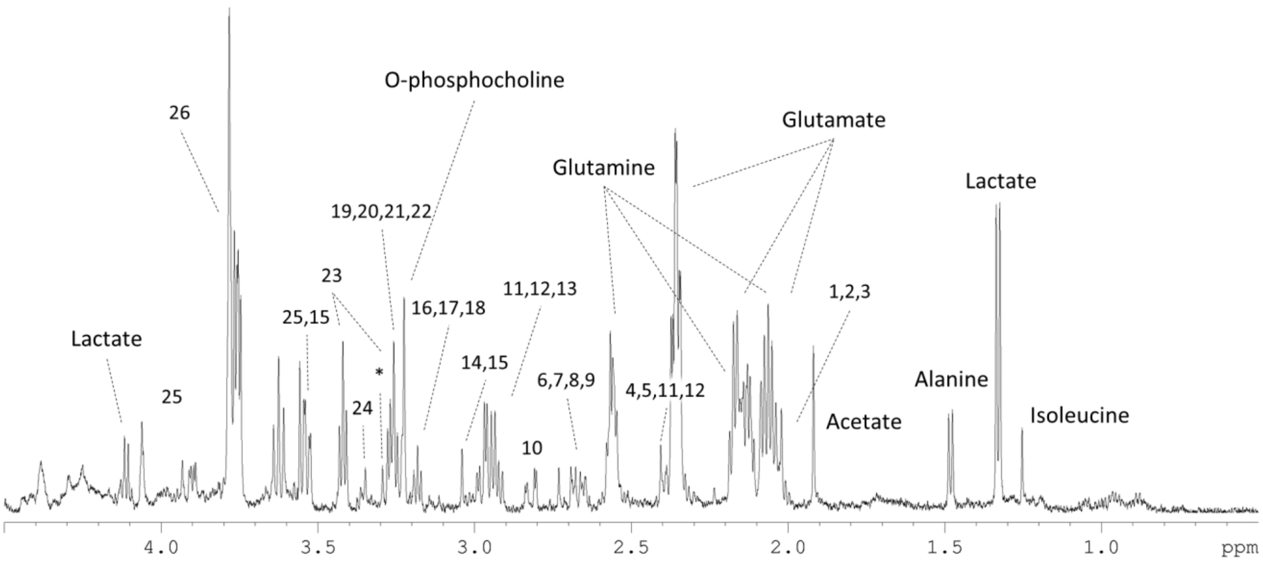

Supplementary Figure 3. 1D ^{1}H NMR spectra of intracellular metabolites from DU145 cells extract. The characteristic peaks from most of the identified metabolites for this cell line are annotated with names and numbers: **1** N-acetylaspartate, **2** N-acetylglutamine, **3** Proline, **4** Pyruvate/Oxaloacetate, **5** Glutathion/Aspargine, **6** Citrate, **7** Methionine, **8** Hypotaurine, **9** Malate, **10** Aspartate, **11** Lysine, **12** α-ketoglutarate, **13** Glutathion, **14** Creatine, **15** Phosphocreatine, **16** Phenylalanine, **17** Histidine, **18** Choline, **19** Taurine, **20** Betaine, **21** Trimethylamine-N-oxide, **22** Myoinositol, **23** Taurine, **24** D-glucose, **25** Myoinositol, **26** Overlapped peaks from D-glucose, D-galactose, Fructose, GSH and several amino acids (-CαH-), * Methanol (residual extraction solvent).
